# Is Increased Resting Heart Rate after Radiofrequency Pulmonary Vein Isolation a Predictor of Favorable Long-Term Outcome of the Procedure?

**DOI:** 10.3390/jcm11082159

**Published:** 2022-04-12

**Authors:** Cezary Maciejewski, Michał Peller, Piotr Lodziński, Edward Koźluk, Agnieszka Piątkowska, Dariusz Rodkiewicz, Izabela Sierakowska, Natalia Roman, Diana Wiśniewska, Dominika Żółcińska, Dominika Rymaszewska, Grzegorz Opolski, Marcin Grabowski, Paweł Balsam

**Affiliations:** 1st Chair and Department of Cardiology, Medical University of Warsaw, 02-091 Warszawa, Poland; cmaciejewski6@gmail.com (C.M.); piotr.lodzinski@me.com (P.L.); ekozluk@vp.pl (E.K.); agnes.piatkowska@wp.pl (A.P.); rodkiewicz@gmail.com (D.R.); sierakowska.izabela@gmail.com (I.S.); nataliaroman72@gmail.com (N.R.); dianabeatawisniewska@gmail.com (D.W.); dominikazolcinska@interia.pl (D.Ż.); dmnrymaszewska@gmail.com (D.R.); grzegorz.opolski@gmail.com (G.O.); grabowski.marcin@me.com (M.G.); pawel.balsam@me.com (P.B.)

**Keywords:** heart rate, pulmonary vein isolation, ganglionated plexi, autonomic nervous system

## Abstract

Background: Increased resting heart rate (RHR) after pulmonary vein isolation (PVI) for treatment of atrial fibrillation (AF) is a common observation, possibly resulting from ganglionated plexus modification during ablation. Previous trials have suggested that an increase in RHR after ablation might be related to higher efficacy of the procedure. The aim of this study was to determine whether or not higher increase in RHR after radiofrequency (RF) PVI might predict better long-term outcome of the procedure in a real-life cohort of patients in whom index ablation for paroxysmal AF was performed. Material and methods: The health records of patients who underwent index point-by-point or drag lesion RF PVI for paroxysmal AF in our department between January 2014 and November 2018 were analyzed. Resting heart rate (RHR) was determined from 12-lead ECG recorded prior to the ablation and before discharge to evaluate changes in RHR after PVI. Only patients in sinus rhythm before the procedure and at discharge were included in the analysis. Telephone follow-up was collected for evaluation of arrhythmia recurrence status. Results: A total of 146 patients who underwent PVI for paroxysmal AF were included. Mean follow-up time was 3.5 years. RHR increased from 64 [58.5–70], prior to procedure, to 72 [64.25–80] bpm at discharge (*p* < 0.001). Higher increase in RHR was not protective from arrhythmia recurrence in long-term observation in both univariable HR = 1.001 (CI 0.99–1.017, *p* = 0.857) and multivariable analyses HR = 1.001 (CI 0.99–1.02, *p* = 0.84). Conclusions: RHR after PVI increased in comparison to baseline in our cohort. However, we did not observe higher increase in RHR to be associated with more favorable long-term effectiveness of the procedure.

## 1. Introduction

Pulmonary vein isolation (PVI) is the most effective treatment method in patients with symptomatic atrial fibrillation (AF) [1]. The estimated success rate of the procedure varies from 47% to 87% [2]. Advanced age, obstructive sleep apnoea, obesity, hypertension, diabetes, reduced left ventricular ejection fraction, long history of AF, left atrium (LA) fibrosis and its enlargement, and thyroid disease, are widely recognized risk factors for arrhythmia recurrence after PVI [3]. Accumulation of these clinical risk factors substantially increases the risk of AF recurrence post-PVI [4].

New predictors of the long-term effectiveness of PVI are currently the subject of research. Previous studies have suggested that acceleration of the resting sinus rhythm after ablation, possibly due to modification of the cardiac autonomic nervous system, may predict a favorable long-term outcome of the procedure. In particular, an increase in resting heart rate (RHR) over 15 beats per minute (bpm) has been suggested by some studies as an indicator of improved prognosis [5,6].

Change in RHR post-ablation could be an easy-to-use parameter in everyday clinical practice; it could inform about the efficacy of the procedure, and is therefore worth investigating. The aim of this study was to determine whether the degree of RHR increase after PVI is an independent predictor of favorable long-term effectiveness of the procedure.

## 2. Materials and Methods

In this single-center, retrospective study, we included patients ≥ 18 years of age with paroxysmal atrial fibrillation, admitted for index PVI between January 2014 and March 2019 to the department of cardiology of a tertiary care teaching hospital. In all patients, radiofrequency, point-by-point or drag lesion pulmonary vein isolation was performed. All participants had 12-lead ECG performed prior to ablation and at discharge from the hospital. ECG was taken after a minimum of 2 min of rest.

Review of medical records was performed to identify: baseline characteristics; medications prior to admission and at discharge; details of ablation procedure and perioperative period; blood tests results; and RHR registered at the beginning of the procedure and at discharge from the hospital based on mean heart rate from 10 s 12-lead ECG tracings. Changes in dosing regimen of amiodarone, class I antiarrhythmics, sotalol and beta-blockers prior- and post-PVI were evaluated and divided into three categories: (1) no change in drug/dosing; (2) initiation or increase in dosing; (3) withdrawal or decrease in dosing. Subsequently, during the period of April 2019 to May 2020, telephone follow-up of patients was performed to evaluate freedom from AF after the procedure. Comparisons of baseline characteristics, pharmacotherapy pre- and post-PVI, and long-term effectiveness of PVI between groups were performed. The analyzed study end-point was AF recurrence after PVI. Patients were divided into two groups using RHR increase of over 15 beats per minute (bpm) as a cutoff point based on the evidence from prior studies [5,6,7]. We sought to determine independent predictors of AF recurrence, including RHR increase.

Due to the retrospective character of the study, the approval of a local ethics committee was waived.

### 2.1. Catheter Ablation Procedure

To exclude the presence of an LA thrombus, patients underwent transesophageal echocardiography within 24 h of the procedure. Point-by-point PVI with the maximal interlesion distance of 6 mm [8] or drag lesion using RF energy was performed. The usual energy settings were 30 watts for 30 s at the anterior LA wall, and 20 to 25 watts at the posterior LA wall. If Ablation Index was used, thresholds of 550 for the anterior wall/roof and 400 elsewhere were used. The pulmonary veins were isolated at the antral level. Procedures were performed in patients under mild sedation, with the goal of obtaining complete PVI. No specific sites of the ganglionated plexi or areas of fractionated potentials were targeted, but other left atrial lines were possible at the operator’s discretion. Additional cavotricuspid isthmus ablation was performed in patients with a concomitant typical atrial flutter.

### 2.2. Statistical Analysis

The results were presented as median and quartiles for continuous and ordinal variables, and as frequencies and percentages for categorical variables. Fisher’s exact test was used for comparison of categorical variables and a Mann–Whitney U test for continuous and ordinal variables. RHR before and after the procedure were compared with the Mann–Whitney U test. Kaplan–Meier survival curves were plotted for groups divided according to RHR increase of ≥15 bpm criterium.

Cox proportional hazards regression analysis was used to identify predictors of atrial fibrillation recurrence. All variables found to be statistically significant in univariable analyses and RHR change were included in the multivariable analysis. A *p* value below 0.05 was considered significant for all tests. For database management and statistical analysis, we used R v.3.6.2.

## 3. Results

During the study period, 197 pulmonary vein isolations for paroxysmal atrial fibrillation were performed. Successful pulmonary vein isolation was obtained in 193 cases, and 160 of the patients were in sinus rhythm both prior to ablation and at discharge. Telephone follow-up was obtained from 146 patients. Mean observation time was 3.47 years (SD 1.91–5.03) post-PVI. AF recurred in 49.3% of patients during follow-up. Baseline characteristics of the study group are presented in Table 1.

Median RHR increased from 64 [58.5–70] bpm prior, to 72 [64.25–80] bpm after PVI (*p* < 0.001) for the entire cohort. The group with RHR increase of ≥15 bpm did not differ significantly from the rest of the cohort in terms of baseline characteristics and pharmacotherapy prior and after PVI (Table 2). 

**Figure 1:**
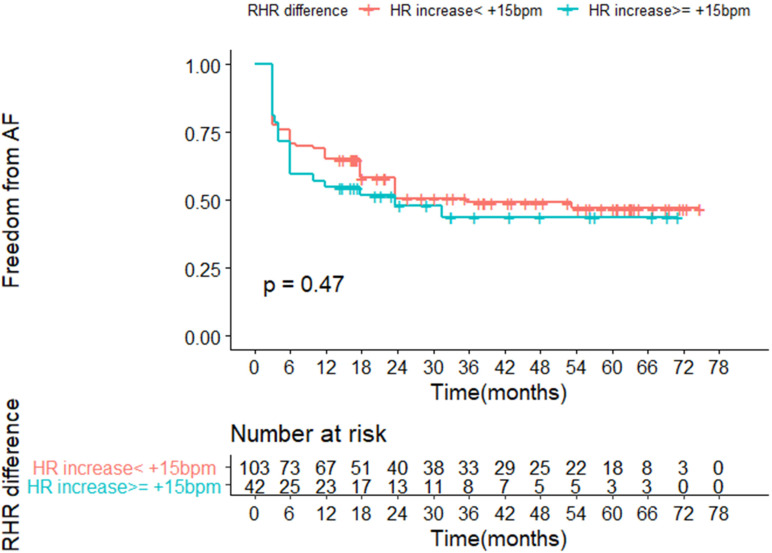
Kaplan–Meier survival curves according to RHR increase of ≥15 bpm criterium; freedom from AF recurrence as the end-point.

Kaplan–Meier survival curves based on RHR increase of ≥15 bpm criterium were plotted (Figure 1). No statistically significant difference in arrhythmia recurrence rate between both study groups was noted (*p* = 0.47).

In a multivariable analysis CHA2DS2VASc score HR = 1.35 (CI 1.11–1.64, *p* = 0.003), the need for cardioversion anytime during hospital stay HR = 2.41 (CI 1.34–4.32, *p* = 0.003), and initiation/increased dosage of beta-blockers after PVI HR = 2.07 (CI 1.08–3.95, *p* = 0.028), were independent predictors of arrhythmia recurrence (Table 3). Increase in RHR was not protective from arrhythmia recurrence in either univariable HR = 1.001 (CI 0.99–1.017, *p* = 0.857) (Table 4) or multivariable analysis HR = 1.001 (CI 0.99–1.02, *p* = 0.84) (Table 3).

## 4. Discussion

The main aim of the presented study was to examine the relation between the degree of RHR increase after RF-PVI and the long-term effectiveness of the RF-PVI procedure. Although increased RHR was a common observation in the analyzed group, it was not associated with a lower rate of arrhythmia recurrence. This result was confirmed in both univariable and multivariable analyses. The CHA2DS2VASc score and the need for cardioversion anytime during hospital stay were the strongest, independent predictors of AF recurrence. These findings were not surprising, as CHA2DSVASc and early AF recurrence post-PVI are known risk factors for arrhythmia recurrence [9,10].

The role of the autonomic nervous system (ANS) in the pathophysiology of atrial fibrillation has been extensively researched [3]. The intrinsic part of the heart’s ANS is located primarily in the ganglionated plexi (GP). The GP contain primarily parasympathetic, but also sympathetic neurons. Hyperactive GP were proposed as one possible mechanism of arrhythmia induction and perpetuation [11]. Four out of the seven GP are located around the pulmonary veins and the left atrium junction [12,13]—the location where PVI is performed. Therefore, inadvertent ablation of the GP is possible. Increase in heart rate after PVI is a common observation, long-described in electrophysiology literature, and likely resulting from modification of the ANS located in the GP [14,15].

Targeted ablation of the GP was one of the techniques proposed for improvement of long-term effectiveness of the procedure [16]. Some randomized control studies prospectively examined the impact of deliberate GP ablation as an adjunct to conventional PVI, but yielded conflicting results [17,18]. Varying ablation techniques, different AF type (paroxysmal or persistent), and distinct methods used for successful GP ablation confirmation might have been the reason for inconsistent findings. Therefore, whether or not ablation of GP positively impacts the outcomes of AF ablation remains uncertain [3].

In our study, we hypothesized that patients with significantly increased RHR after PVI would be those in whom unwilling GP modification occurred which could translate into more favorable efficacy of the procedure in long-term observation. Several previous studies have investigated such an hypothesis, reporting different findings.

Goff, et al. [6] in a retrospective study, analyzed the association between heart rate increase post-PVI and freedom from AF at 1-year in a group of 257 patients. PVI was performed by radiofrequency or cryoballoon ablation. All patients had a standard 12-lead ECG performed while awake prior to PVI, and the morning after the procedure. Only patients with paroxysmal AF in sinus rhythm prior to PVI and the morning after were selected for the study. The average HR increased from 60.6 ± 11.3 bpm prior to PVI, to 70.7 ± 12.0 bpm post-PVI. Patients with recurrence of AF had lower post-PVI HR than those who remained free from AF (67.8 ± 10.2 vs. 73.3 ± 13.0 bpm; *p* < 0.001). The probability of AF recurrence at 1-year decreased as the change in HR increased (estimated OR 0.83; 95% CI [0.74–0.93]; *p* = 0.002). HR increase > 15 bpm was associated with the lowest odds of AF recurrence (estimated OR 0.39; 95% [0.17–0.85]; *p* = 0.018) compared to HR decrease.

In another study, Sikorska, et al. [5] analyzed the post-ablation RHR increase as a predictor of freedom from arrhythmia recurrence at 1-year observation. The study included a cohort of 111 patients in whom index PVI (RF or cryoballoon) for paroxysmal AF was performed. Patients were in sinus rhythm before and after ablation. RHR was calculated from a standard 12-lead ECG recorded one day before, and two days after, ablation. Increase in RHR of 15.1 bpm (SD 10.4) was observed among the responders to ablation, while the group of nonresponders to the procedure was characterized by a mean increase in RHR of 10.7 (SD 9) bpm (*p* < 0.0003). There were no significant differences in RHR change and ablation efficacy regarding method of PVI (cryoballoon or RF). A multivariable analysis demonstrated RHR increase after ablation to be an independent predictor of freedom from arrhythmia recurrence at 1-year post-procedure (OR 1.06, CI: 1.00–1.12, *p* = 0.04). RHR increase of ≥15 bpm predicted the ablation success with the highest total accuracy of 59.5% (sensitivity 52.7%, specificity 73%).

Maj, et. al. [7] evaluated changes of RHR after cryoballoon PVI as a predictor of long-term outcome of the procedure. At 2-year follow-up, freedom from recurrences was 83.1% for the patients with HR increase ≥ 15 bpm after CB-PVI, and 66.3% in patients with HR increase ˂ 15 bpm (*p* = 0.021), therefore suggesting a more favorable outcome in individuals with higher RHR increase. However, RHR increase was not found to be an independent predictor of ablation success in multivariable analysis (adjusting for age, LA dimension and type of AF).

In a prospective study, Kuyumcu, et al. [19] documented an increase of RHR after cryoballon PVI for paroxysmal AF. No difference in the degree of RHR increase was noted in patients with and without recurrence in the 3-month follow-up period. The study did not evaluate the outcome of PVI in long-term observation.

The aforementioned evidence shows that there is no unequivocal data on whether an increase in RHR can be considered as an indicator of higher AF ablation efficacy. The results of these studies are summarized in Table 5.

Mean follow-up in our study was 3.5 years, which is significantly longer than in most previous publications. This allowed for comparison of long-term freedom from AF. In addition, to our best knowledge, our study is the first to present a cohort consisting solely of patients who underwent RF PVI. This makes our cohort more homogenous in comparison to previous studies which recruited patients in whom either RF or cryoballoon PVI was performed.

### Limitations

There are some limitations that must be considered. Small cohorts of patients could have been insufficient to demonstrate a statistically significant difference in AF recurrence regarding change of RHR. Single ECG tracings performed before and after PVI might have been insufficient to detect changes in RHR accurately–nevertheless investigation of such an easy-to-use-in-everyday-clinical-practice parameter was worth pursuing, and was the purpose of the analysis. Although all patients had radiofrequency PVI performed, the methods varied: drag lesion or point-by-point PVI guided with contact force or ablation index. The follow-up was collected through telephone directly from patients, therefore arrhythmia recurrence status was not confirmed by ECG recordings. This might have led to some degree of underestimation of arrhythmia recurrence although it should not have had a negative impact on the main purpose of our analysis, as the same follow-up methodology was used for all patients, irrespective of observed RHR changes.

## 5. Conclusions

This study was performed to evaluate the clinical observation that resting HR increases after PVI and that the degree of increase in outcome is linked to ablation success.

Our analysis confirmed that RHR increases in most patients following the RF PVI. However, the degree of RHR acceleration was not an indicator of more favorable long-term effectiveness of the procedure.

## Figures and Tables

**Table 1:** Baseline characteristics.

Variable	N = 146
Age, years, median [IQR]	60 [52–66]
Females, n (%)	56 (39.2%)
Heart failure	2 (1.4%)
Hypertension	85 (58.6%)
Coronary artery disease	14 (9.6%)
Vascular disease	4 (2.8%)
Diabetes mellitus	17 (11.7%)
Smoking history	36 (24.6%)
CHA_2_DS_2_-VASc score, median [IQR]	1.5 [1–2] *140*
Thyroid disease	18 (12.3%)
Additional CTI ablation	6 (4.1%)
Any additional lines except CTI ablation	21 (14.4%)
BMI	27.75 [25.8–30.3] *106*
Family history of AF	21 (14.5%)
Cardioversion anytime during hospital stay	24 (16.44%)
eGFR	60 [60–60] *122*
RBC, median [IQR]	4.75 [4.44–5.04] *106*
RHR before PVI, median [IQR]	64 [58.5–70]
RHR before PVI, mean [SD]	65.4 [13.5]
RHR post PVI, median [IQR]	72 [64.25–80]
RHR post PVI, mean [SD]	72 [10.9]
Pharmacotherapy prior PVI	
Beta-blockers	96 (66.2%)
Amiodarone	13 (9%)
Class I Antiarrhythmic drugs	55 (37.9%)
Sotalol	21 (14.5%)
Pharmacotherapy after PVI	
Beta-blockers	108 (74%)
Amiodarone	0 (0%)
Class I Antiarrhythmic drugs	10 (6.9%)
Sotalol	4 (2.7%)
Recurrence of AF during follow up	72 (49.3%)

Abbreviations: eGFR, estimated glomerular filtration rate; CTI, cavotricuspid isthmus; IQR, interquartile range; RBC, red blood cells count; Number provided in italic indicates the total number of patients available for the variable if there are some missing data.

**Table 2:** Baseline characteristics of the groups according to change in RHR.

Variable	RHR Change-Rest of the Cohort N = 103	RHR Change-Increase ≥ 15 bpm N = 43	*p* Value
Age, years, median [IQR]	60 [50–66]	60 [53–66]	0.98
Females, n (%)	41 (41%)	15 (34.9%)	0.58
Heart failure	2 (1.9%)	0 (0%)	1
Hypertension	60 (58.8%)	25 (58.1%)	1
Coronary artery disease	11 (10.68%)	3 (6.98%)	0.76
Vascular disease	3 (2.9%)	1 (2.38%)	1
Diabetes mellitus	11 (10.78%)	6 (13.95%)	0.58
Smoking history	25 (24.27%)	11 (25.58%)	1
CHA_2_DS_2_-VASc score, median [IQR]	2 [1–2] *98*	1 [1–2.75] *42*	0.74
Thyroid disease	14 (13.59%)	4 (9.30%)	0.59
Additional CTI ablation	4 (3.88%)	2 (4.65%)	1
Any additional lines except CTI ablation	18 (17.48%)	3 (6.98%)	0.124
BMI, median [IQR]	28.4 [25.8–30.8] *78*	27.1 [25.6–29.5] *28*	0.35
Family history of AF	16 (16.49%)	5 (12.2%)	0.61
Cardioversion anytime during hospital stay	18 (17.48%)	6 (13.95%)	0.81
eGFR	60 [59.8–60] *87*	60 [60–71.5] *35*	**0.027**
RBC, median [IQR]	4.77 [4.43–5.03] *78*	4.60 [4.30–4.88] *28*	0.257
Pharmacotherapy prior PVI			
Beta-blockers	67 (65.7%)	29 (67.44%)	1
Amiodarone	8 (7.84%)	5 (11.63%)	0.53
Class I Antiarrhythmic drugs	38 (37.25%)	17 (39.53%)	0.85
Sotalol	16 (15.69%)	5 (11.63%)	0.61
Pharmacotherapy after PVI			
Beta-blockers	76 (73.79%)	32 (74.41%)	1
Amiodarone	0 (0%)	0 (0%)	1
Class I Antiarrhythmic drugs	8 (7.67%)	2 (4.65%)	0.72
Sotalol	3 (2.91%)	1 (2.33%)	1
Recurrence of AF during follow up	50 (48.54%)	22 (51.16%)	0.87

Abbreviations: eGFR, estimated glomerular filtration rate; CTI, cavotricuspid isthmus; IQR, interquartile range; RBC, red blood cells count; Number provided in italic indicates the total number of patients available for the variable in case of missing data.

**Table 3:** Multivariable analysis of predictors of AF recurrence.

Variable	Primary Endpoint		
	HR	95% CI	*p*-Value
HR increase (bpm)	1.001	0.99–1.02	0.84
CHA2DS2VASc	1.35	1.11–1.64	**0.003**
Cardioversion anytime during hospital stay	2.41	1.34–4.32	**0.003**
Beta blockers change (no change in dosage as reference)			
Withdrawal or decrease in dosage	1.36	0.55–3.43	0.50
Initiation or increase in dosage	2.07	1.08–3.95	**0.028**

**Table 4:** Univariable analyses of predictors of AF recurrence.

Variable	Primary Endpoint		
	HR	95% CI	*p*-Value
Delta HR	1.001	0.99–1.017	0.85
Age, years, median	1.02	0.99–1.04	0.08
Female sex	1.58	0.99–2.53	0.054
Heart failure	2.82	0.70–11.6	0.15
Hypertension	1.19	0.74–1.91	0.47
Coronary artery disease	1.77	0.90–3.45	0.10
Vascular disease	1.02	0.25–4.15	0.98
Diabetes mellitus	1.39	0.71–2.71	0.33
Smoking history	1.06	0.63–1.80	0.82
CHA_2_DS_2_-VASc score	1.31	1.08–1.58	**0.006**
Thyroid disease	1.11	0.55–2.23	0.77
Additional cavotricuspid isthmus ablation	1.83	0.67–5.01	0.24
Any additional lines except cavotricuspid isthmus ablation	1.35	0.74–2.47	0.32
BMI	1.02	0.96–1.09	0.55
Family history of AF	0.70	0.35–1.42	0.33
Cardioversion anytime during hospital stay	1.80	1.03–3.14	**0.04**
eGFR	1.004	0.98–1.02	0.72
AF duration since first documented AF (months)	0.999	0.99–1.004	0.94
Amiodarone change			
Withdrawal or decrease in dose	1.72	0.82–3.59	0.15
Initiation or increase in dose	NA	NA	NA
Antiarrhythmic class I change			
Withdrawal or decrease in dose	0.63	0.37–1.09	0.10
Initiation or increase in dose	NA	NA	NA
Sotalol change			
Withdrawal or decrease in dose	1.03	0.51–2.07	0.93
Initiation or increase in dose	3.82	0.52–27.97	0.19
Beta blockers change			
Withdrawal or decrease in dose	1.28	0.51–3.20	0.60
Initiation or increase in dose	2.11	1.17–3.81	**0.01**

**Table 5:** Summary of the results of the studies evaluating the relationship between RHR change and PVI efficacy.

Study	Number of Patients	Pre-PVI RHR, bpm (Mean, SD)	Post-PVI RHR, bpm (Mean, SD)	Freedom from AF at 1-Year
Goff, et al.	257	60.6 [11.3]	70.7 [12.0]	52%
Sikorska, et al.	111	57 [8.8]	71.4 [11.1]	67%
Maj, et al.	472	60.2 [10.4]	75.5 [12.0]	NA
Kuyumcu, et al.	45	82.5 [15.23]	85.5 [11.16]	NA
Maciejewski, et al.	146	65.4 [13.5]	72 [10.9]	62.3%

## Data Availability

The study did not report any data.
